# PXL01 in Sodium Hyaluronate for Improvement of Hand Recovery after Flexor Tendon Repair Surgery: Randomized Controlled Trial

**DOI:** 10.1371/journal.pone.0110735

**Published:** 2014-10-23

**Authors:** Monica E. Wiig, Lars B. Dahlin, Jan Fridén, Lars Hagberg, Sören E. Larsen, Kerstin Wiklund, Margit Mahlapuu

**Affiliations:** 1 Department of Surgical Science, Hand Surgery, Uppsala University, Uppsala, Sweden; 2 Uppsala University Hospital, Uppsala, Sweden; 3 Department of Clinical Sciences Malmö - Hand Surgery, Lund University, Skåne University Hospital, Malmö, Sweden; 4 Department of Hand Surgery, Sahlgrenska University Hospital, Göteborg, Sweden; 5 Department of Hand Surgery, Department of Clinical Science and Education, Södersjukhuset, Karolinska Institutet, Stockholm, Sweden; 6 Department for Orthopaedics, Unit for Hand Surgery, Odense University Hospital, Odense, Denmark; 7 Pharma Consulting Group Solutions AB, Uppsala, Sweden; 8 Pergamum AB, Stockholm, Sweden; University of Insubria, Italy

## Abstract

**Background:**

Postoperative adhesions constitute a substantial clinical problem in hand surgery. Fexor tendon injury and repair result in adhesion formation around the tendon, which restricts the gliding function of the tendon, leading to decreased digit mobility and impaired hand recovery. This study evaluated the efficacy and safety of the peptide PXL01 in preventing adhesions, and correspondingly improving hand function, in flexor tendon repair surgery.

**Methods:**

This prospective, randomised, double-blind trial included 138 patients admitted for flexor tendon repair surgery. PXL01 in carrier sodium hyaluronate or placebo was administered around the repaired tendon. Efficacy was assessed by total active motion of the injured finger, tip-to-crease distance, sensory function, tenolysis rate and grip strength, and safety parameters were followed, for 12 months post-surgery.

**Results:**

The most pronounced difference between the treatment groups was observed at 6 months post-surgery. At this timepoint, the total active motion of the distal finger joint was improved in the PXL01 group (60 *vs.* 41 degrees for PXL01 *vs.* placebo group, p = 0.016 in PPAS). The proportion of patients with excellent/good digit mobility was higher in the PXL01 group (61% *vs.* 38%, p = 0.0499 in PPAS). Consistently, the PXL01 group presented improved tip-to-crease distance (5.0 *vs.* 15.5 mm for PXL01 *vs.* placebo group, p = 0.048 in PPAS). Sensory evaluation showed that more patients in the PXL01 group felt the thinnest monofilaments (FAS: 74% *vs.* 35%, p = 0.021; PPAS: 76% *vs.* 35%, p = 0.016). At 12 months post-surgery, more patients in the placebo group were considered to benefit from tenolysis (30% *vs.* 12%, p = 0.086 in PPAS). The treatment was safe, well tolerated, and did not increase the rate of tendon rupture.

**Conclusions:**

Treatment with PXL01 in sodium hyaluronate improves hand recovery after flexor tendon repair surgery. Further clinical trials are warranted to determine the most efficient dose and health economic benefits.

**Trial Registration:**

ClinicalTrials.gov NCT01022242; EU Clinical Trials 2009-012703-25.

## Introduction

Postoperative adhesions are fibrous tissue connections forming when the body's repair mechanisms respond to surgical trauma or other types of tissue injury. General abdominal, vascular, gynaecological, urological, and orthopaedic surgery may lead to adhesion formation in up to 95% of the patients [1]–[3]. Adhesions after abdominal and pelvic surgery may cause small bowel obstruction, female infertility, as well as an increased risk of intra- and postoperative complications and prolonged operative time [4]. In the field of hand surgery, the formation of adhesions between the tendon and tendon sheath or adjacent tissues after flexor tendon injury and repair, restricts the gliding function of the tendon, ultimately resulting in decreased mobility of the affected digit and impaired postoperative recovery of the hand function [5]. This is recognized as a particular problem for injuries in zones I and II of the hand (the volar side of the fingers), where the tendon excursion relative to the tendon sheath is the largest, and therefore, peritendinous adhesions have the highest impact on finger mobility [6]. The current best practice is designed to avoid adhesion formation by means of careful surgical technique, causing minimal trauma, combined with early mobilisation of the hand. Nonetheless, reduction in post-surgical mobility of the injured finger frequently leads to severe social and economic consequences both for the patient and society, such as prolonged sick leave [7], [8]. In average, flexor tendon repairs may require a secondary surgical procedure to remove the adhesions, e.g. tenolysis, in one out of four cases [9]. Thus, there is a strong medical demand supporting the need to develop pharmaceutical products for prevention of peritendinous adhesions in connection to hand surgery.

The current study was conducted in order to evaluate the efficacy and safety of local administration of PXL01, formulated in viscous solution of sodium hyaluronate, in preventing adhesion formation, and correspondingly improving hand function, in connection to flexor tendon repair surgery after injury. PXL01 is a synthetic peptide sequentially derived from human lactoferrin, an iron-binding glycoprotein present in milk and mucosal secretions, which exhibits antimicrobial and anti-inflammatory properties [10], [11]. *In vitro* studies in human cell lines have shown that PXL01 exhibits an inhibitory effect on the most important hallmarks of adhesion formation by reducing secretion of inflammatory cytokines, promoting fibrinolysis and reducing infections [12]. These pharmacological activities of PXL01 are combined with the lubricating properties of the carrier sodium hyaluronate, which acts as an initial diffusion barrier for the fibrinogen exudates and also allows PXL01 to be slowly released [12]. In recent nonclinical studies, PXL01 with sodium hyaluronate as a carrier was demonstrated to reduce post-surgical adhesions in experimental models of abdominal surgery in rats [12] and flexor tendon repair surgery in rabbits [13], [14]. Importantly, in these studies no negative effect of PXL01 on healing was seen by assessing the force needed for failure of bowel anastomosis in rats or of the repaired tendons in rabbits. A first-in-man, phase I, single-blind, placebo-controlled study investigating local tolerability, safety and pharmacokinetics in three doses of PXL01 and placebo, has been performed in 15 healthy male volunteers [15]. A dose of 10, 20 or 40 mg of PXL01 in sodium hyaluronate or placebo (sodium hyaluronate) was administered by single abdominal subcutaneous injection. No findings of concern related to the local tolerability or safety were reported. The systemic exposure of PXL01 was low (below 100 ng/ml), suggesting that in connection to local application only a small fraction of the peptide reaches the bloodstream. Based on this combined evidence of efficacy and safety of the compound, the current study investigated the effect of single treatment of PXL01 in sodium hyaluronate on recovery of hand function for up to 12 months after surgical flexor tendon repair, following injury in zones I or II of the hand.

## Methods

### Ethics statement

This study was approved by the Regional Ethical Review Board in Uppsala, Sweden; by the Regional Ethical Review Board in Hillerød, Denmark, and by the Regional Ethical Review Board in Düsseldorf, Germany, and it adhered to the Declaration of Helsinki guidelines. Written, informed consent was obtained from each participant. The protocol for this trial and supporting CONSORT checklist are available as supporting information; see Protocol S1 and Checklist S1.

### Overall study design

This was a multi-centre, randomised, parallel group study evaluating the efficacy and safety of peptide PXL01 compared to placebo in patients admitted for flexor tendon repair surgery after injury in zones I or II of the hand. The study took place in hand surgery clinics in Sweden, Denmark and Germany. Eligible patients were randomly allocated to 1 of the 2 treatment groups: PLX01 or placebo (1∶1), in a sequential fashion. Only 1 digit was to be treated. Patients were administered PXL01 (0.5 ml of 20 mg/ml) in viscous gel of sodium hylarunate (15 mg/ml) or placebo (0.5 ml of a 9 mg/ml sodium chloride solution), locally between the flexor tendon and the tendon sheath, and around the tendon sheath, following surgical repair of the flexor tendon, prior to closure of the surgical wound. Each patient was to follow 1 of 2 post-operative mobilisation programs: Kleinert mobilisation with active hold or active mobilisation. All patients at 1 centre followed the same mobilization program as decided by each centre. All the patients received the detailed training program and instructions. The study comprised 9 visits. Screening, surgery and investigational medicinal product (IMP) administration were performed on the same day (Day 0) at Visit 1. The patients returned to the clinic for efficacy and safety assessments 1 to 5 days, 2, 4, 6, 8, 12 weeks, and 6 and 12 months after surgery/IMP administration.

A hand surgery specialist, or a non-specialist with experience of at least 5 individually performed flexor tendon repairs in zones I or II, performed tendon surgery and product administration. Trained rehabilitation personnel performed the measurements of post-surgical hand mobility. This was a double-blind study. However, due to the difference in viscosity between the PXL01 and placebo solutions, the surgeons may not have been blinded after mixing the respective IMP components and applying the IMP. The patients and the rehabilitation personnel evaluating the outcome measrues were, however, blinded throughout the study.

### Drug substance, drug product and carrier

The drug substance PXL01 acetate was manufactured at Bachem AG, Bubendorf, Switzerland using solid phase peptide synthesis. The drug product - PXL01 50 mg/ml concentrate for solution for injection - was manufactured at Apoteket Production & Laboratories AB, Umeå, Sweden by dissolving PXL01 acetate in 0.9% sodium chloride solution, followed by filter sterilization. The solvent - sodium hyaluronate 25 mg/ml (molecular weight 1.5 to 8.1×10^6^ Da) - was manufactured at Bohus BioTech AB, Strömstad, Sweden by dissolving sodium hyaluronate fibre derived from rooster combs in 0.9% sodium chloride solution, followed by steam sterilization. Prior to administration, PXL01 concentrate was diluted in sodium hyaluronate solvent in the operating theatre. The concentration of the components after dilution was 20 mg/ml of PXL01 and 15 mg/ml of sodium hyaluronate. 0.5 ml of the mixed product was administered in the surgical area in flexor tendon repair surgery, corresponding to a dose of 10 mg PXL01.

### Study population

Patients aged between 12 and 75 years with an open flexor tendon injury, characterised by a complete division of the flexor digitorum profundus (FDP) tendon in zones I or II, with or without division of the flexor digitorum superficialis (FDS), and possible to rejoin with tendon suture, were considered eligible to participate in the study. The flexor tendon injury was to be operated and sutured within 14 days after trauma. Thumb injuries, joint or bilateral injures, concomitant fracture(s) and palmar plate injuries requiring immobilization of the finger/hand, injuries with associated soft tissue loss or requiring vascular repair, as well as severe crash injuries were excluded from the trial. Subjects with reduced motion of the digit, which was to be treated with IMP, or the corresponding contralateral digit, prior to the injury, were excluded from the trial.

### Study endpoints

All efficacy assessments were performed on the treated digit as well as on the contralateral digit unless otherwise stated. Preferably, the same person was to evaluate the patients at the different visits.

The flexion and extension at the metacarpophalangeal (MCP), proximal interphalangeal (PIP) and distal interphalangeal (DIP) joints were measured on the dorsal side of the fingers using the goniometer (Patterson Medical, UK) at visits performed 4, 6, 8, 12 weeks, and 6 and 12 months post-surgery (see Fig. S1A, B). The sum of flexion at the PIP and the DIP joint in attempt fist position minus the extensor lag at these joints was used to compute total active motion 2 (TAM2). The sum of flexion at the MCP, PIP and DIP joint in attempt fist position minus the extensor lag at these joints was used to compute TAM3. Hyperextension was to equal 0 degrees, when calculating both TAM2 and TAM3. In addition, TAM2 at 12 weeks, and 6 and 12 months post-surgery was graded using the original Strickland's criteria into four functional categories of “excellent” (≥150 degrees), “good” (125 to 149 degrees), “fair” (90 to 124 degrees) or “poor” (<90 degrees) mobility [9]. To measure the total active motion in DIP joint (DIPAM), the DIP flexion was measured with the MCP joint extended and the PIP and DIP joints fully flexed (see Fig. S1C), while the DIP joint extension was measured as described above. DIPAM was estimated as active flexion – extension lag for visits performed 4, 6, 8, 12 weeks, and 6 and 12 months post-surgery. The tip-to-crease distance was measured in mm horizontally from the fingertip to the distal palmar crease (see Fig. S1D) at 4, 6, 8, 12 weeks, and 6 and 12 months post-surgery. Sensory evaluation was performed with Semmes-Weinstein monofilament 5Pc kits (Patterson Medical, UK) on all patients with any complete digital nerve injury at 12 weeks, 6 and 12 months post-surgery. A set of 5 monofilaments was used to assess touch thresholds for pressure (see Table 1 for further details). Each monofilament was applied on each side of the fingertip (ulnar side, radial side or both, depending on the location of the nerve injury) starting with the green monofilament and thereafter in an ascending order until the patient indicated that he/she could feel the pressure. Once a patient felt the pressure of a monofilament, no further monofilaments were tested. At late visits starting from 12 weeks post-surgery, the investigator or delegated study personnel judged whether it was likely that the patient would benefit from tenolysis to improve the mobility in the treated finger. Maximum grip strength was measured using a calibrated JAMAR (Hydraulic Hand Dynamometer, SH5001, SEAHAN Corporation, Korea) at 6 and 12 months post-surgery.

**Table 1:** Interpretation of Semmes-Weinstein monofilaments for sensory evaluation*.

Colour	Sensory function	Filament markings	Calculated force (g)
Green	Normal	1.65 to 2.83	0.0045 to 0.068
Blue	Diminished light touch	3.22 to 3.61	0.166 to 0.408
Purple	Diminished protective sensation	3.84 to 4.31	0.697 to 2.06
Red	Loss of protective sensation	4.56 to 6.65	3.63 to 447
Red-lined	Untestable	>6.65	>447

*Data from [44].

The results of total active motion of the injured finger, tip-to-crease distance and grip strength are presented in the main text as mean values if the data were normally distributed or as median values if the data were skewed distributed. Both mean and median values are presented in descriptive tables and box blots independent if the assumption of normal distributed residuals was fulfilled or not.

In addition, total passive motion (TPM) of the injured finger was assessed. TPM2 and TPM3 were measured as described above for assessment of TAM2/3, with the exception that instead of active motion, the digits were passively moved using the contralateral hand, or with help from the physiotherapist. It is suggested that in case a low mobility in terms of TAM is accompanied by a low mobility in terms of TPM, the limitation in active motion may depend on disabilities and conditions other than adhesions. Therefore, in this study, TPM was measured with the intention to indicate if reductions in TAM for individual patients were related to adhesion formation or to other concomitant factors. However, the TPM values for each patient showed large variation between different visits not providing a sound bases to exclude any patient from further analysis (data not shown).

Safety was followed up immediately post-surgery [adverse events (AEs), vital signs, clinical chemistry and haematology], 1 to 5 days (AEs), 2 weeks (AEs, vital signs, clinical chemistry, haematology, examination of the surgical area and rate of tendon rupture) and 4, 6, 8, 12 weeks, and 6 and 12 months after surgery/IMP administration (AEs and rate of tendon rupture). Only AEs relating to the surgical area and AEs suspected to be probably or possibly related to the IMP were registered from the visit performed at 12 weeks post-surgery/IMP administration and up until the final visit at 12 months post-surgery. Serious adverse events (SAEs) were registered from the time of IMP administration until the final visit at 12 months post-surgery. The surgical area of the treated digit was examined in terms of wound healing (normal, suture rupture, granulation tissue, other), scar appearance (normal, widened, hypertrophic, keloid, induration, other) and signs of infection (yes/no, if “yes” the diagnoses were to be reported as AEs). In case the study personnel judged a tendon rupture was verified in any of the injured digits, the patient was to be withdrawn from the study.

### Sample size

The size of the study population was calculated to show a minimal difference in TAM2 of 20 degrees. The standard deviation of TAM2 was estimated from a database including patients with flexor tendon injuries in zone II, who had undergone tendon repair surgery at the University Hospital in Uppsala (one of the clinical sites in this trial) between years 2000 and 2006. With an estimated standard deviation of 37 degrees (equal in both treatment groups), 110 subjects were needed (55 in each treatment group) to assure 80% power to detect a difference of 20 degrees in TAM2 on the 5% significance level. To adjust for withdrawals, 138 patients were included in this study. A planned interim analysis to provide a basis for a potential recalculation of the required patient number was performed when 69 patients (50%) had completed the visit at 12 weeks post-surgery. Recalculation of the sample size avoiding unblinding was performed as described by A.L. Gould [16]. The results indicated that the variation was not higher than expected; hence, no adjustment of the number of patients was necessary.

### Statistical analysis

All statistical calculations were performed using SAS versions 9.2 and 9.3 (SAS Institute Inc., Cary, NC, USA) by Pharma Consulting Group Solutions AB, Uppsala, Sweden. Since the mobility measured at early time points after surgery is not predictive for later values, the last observation carried forward principle was not used to replace missing values for efficacy variables. TAM2, TAM3, DIPAM, tip-to-crease distance and grip strength were tested using analysis of covariance with values of the injured hand as dependent variable and the values of the contralateral hand as covariate. For the parameters where the assumption of normal distributed residuals was not fulfilled, a non-parametric analysis (rank analysis of covariance) was performed. The analysis also included factors for treatment, centre (6 groups) and time between injury and surgery (2 groups: ≤4 days and >4 days). The null hypotheses were that there would be no difference in each of the variables between patients treated with PXL01 and patients treated with placebo for each time point. An exploratory statistical analysis was performed using mixed model repeated measurement analysis (including factors for treatment, centre, time between injury and surgery, days from baseline, and interaction between days from baseline and treatment) for TAM2, TAM3 and DIPAM, up to 12 months post-surgery.

The frequency of recommended tenolysis was analysed using extended Mantel-Haenszel method including treatment, centre (6 groups) and time between injury and surgery (2 groups).

An exploratory statistical analysis (chi-square) of TAM2 values graded according to Strickland's classification was performed, where the original 4 categories were pooled into two functionally relevant groups; excellent/good vs. fair/poor. An additional exploratory statistical analysis of Strickland's classification was performed using generalized estimating equations model, including factors for treatment, centre, days from baseline, time between injury and surgery, and interaction between days from baseline and treatment. An exploratory statistical analysis (chi-square) of sensory evaluation was performed where the five monofilaments were pooled into two functionally relevant groups: green and blue *vs.* purple, red and red-lined monofilaments.

All the reported p values are unadjusted for multiplicity.

## Results

### Disposition of patients and data sets analysed

**Figure 1:**
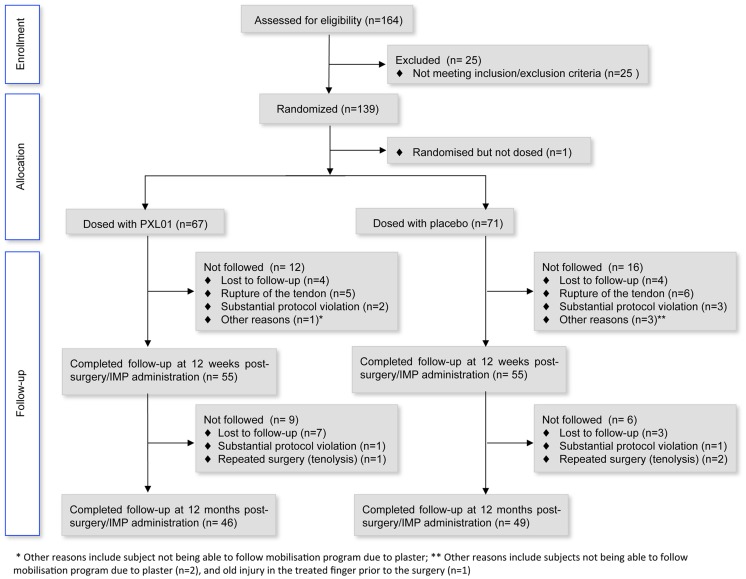
Flow of participants through trial.

A total of 164 patients were screened and 139 were randomised in the study between February 2010 and May 2012, and the last patient completed the final follow-up visit at 12 months post-surgery in February 2013. One ineligible patient was randomised by mistake, but did not receive IMP. At the visit at 12 weeks post-surgery, 55 patients (81%) in the PXL01 group and 55 patients (78%) in the placebo group were still on-going in the study (i.e. had not been withdrawn). The number of patients completing the study at the final visit at 12 months post-surgery was 46 (68%) and 49 (69%) in the PXL01 and placebo groups, respectively. The main reasons for premature withdrawals included: verified rupture of repaired tendon, patient lost to follow-up, substantial protocol violation, repeated surgery (i.e. tenolysis) or a combination of reasons listed above (Fig. 1).

Patients were allocated to each analysis population prior to breaking the blind. The safety population included all randomised patients who had received IMP. The full analysis set (FAS) population included all patients who completed surgery and received IMP, and who did not fail any major eligibility criteria. The per protocol analysis set after 3 months (PPAS3m) and 12 months (PPAS12m) population included patients who had sufficiently complied with the protocol and had available data for analysis of the primary variable at the visit performed 12 weeks and 12 months post-surgery, respectively. In addition, a prerequisite for inclusion in the PPAS12m population was that the patient had been included in the PPAS3m population. All safety analyses were performed on the safety analysis set. All the efficacy analysis up to and including 12 weeks post-surgery were performed on FAS and PPAS3m. All the efficacy analysis of visits at 6 and 12 months post-surgery were performed on FAS and PPAS12m (Table 2).

**Table 2:** Analysis populations*.

Analysis population	PXL01	Placebo	All
Number of randomised patients	68	71	139
Safety population	65 (96%)	70 (99%)	135 (97%)
FAS population	64 (94%)	68 (96%)	132 (95%)
PPAS3m population	41 (60%)	48 (68%)	89 (64%)
PPAS12m population	34 (50%)	43 (61%)	77 (55%)

*Percentages are based on all randomised patients; FAS, full analysis set; PPAS3m, per protocol analysis set after 3 months; PPAS12m, per protocol analysis set after 12 months.

### Baseline characteristics, treatment compliance and concomitant medication

Overall, the demographics and baseline characteristics were comparable between the treatment groups. The mean age of patients was 36 years in both the PXL01 and the placebo group. Similar proportions of males and females were included in both treatment groups (PXL01: 73% males and 27% females, placebo: 72% males and 28% females). A majority of the patients were white (PXL01: 95%, placebo: 91%). The dominant hand was injured in 45% of the patients (PXL01: 44%, placebo: 46%). A majority of the patients in both treatment groups experienced a sharp trauma (PXL01: 92%, placebo: 90%). There were no differences in surgery baseline values, such as incision technique used, technique used for the core suture or epitendinous suture. The demographics and baseline characteristics are summarized in Tables S1, S2.

A single dose of IMP was given to each patient at the clinic during surgery; hence, no additional routines for assessment of IMP compliance were applied. A similar proportion of patients in each treatment group used a majority of the most common concomitant medications.

### Efficacy evaluation

**Figure 2:**
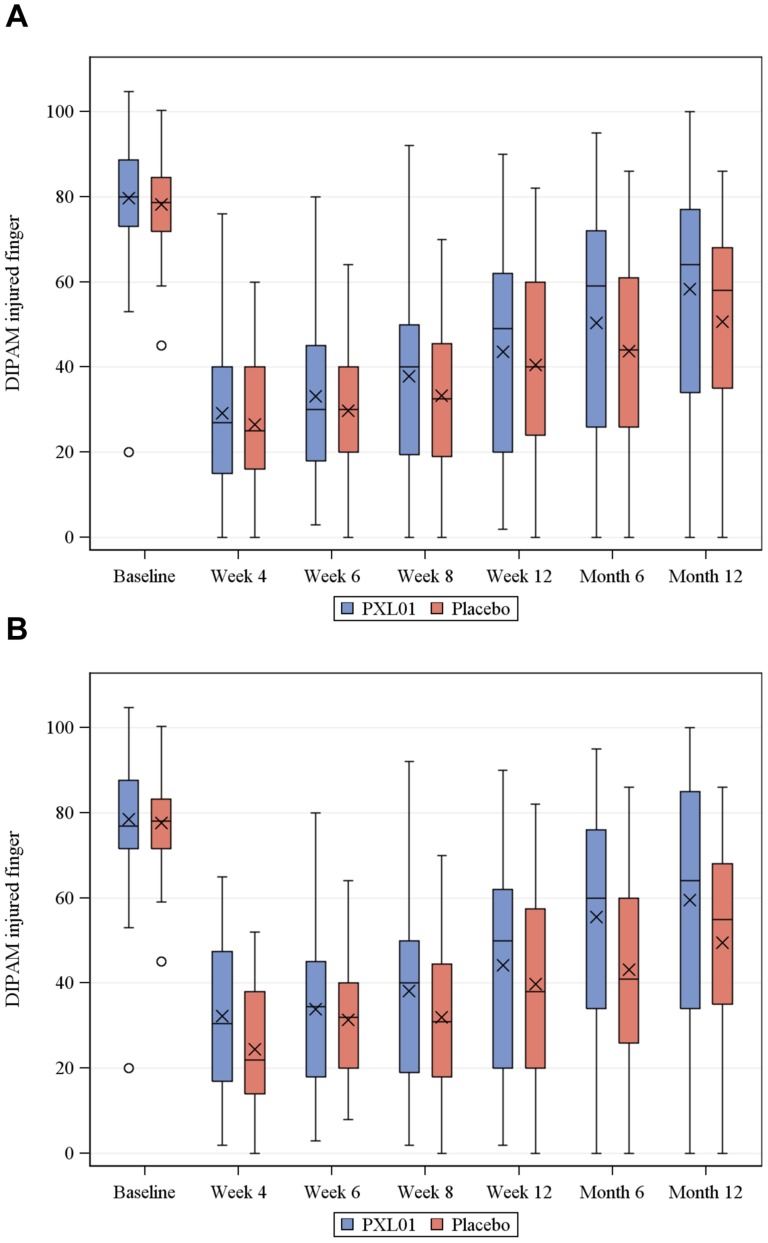
DIPAM of the injured finger over time up to 12 months post-surgery/IMP administration in FAS (A) and PPAS (B). Boxes show the interquartile range that contains values between the 25th and 75th percentile. Crosses (x) denote mean values, whereas lines (−) denote median values. Minimum and maximum values are indicated with bars. Circles denote outliers. At baseline, one outlier of 146 degrees in the PXL01 group is not displayed in the figure. Baseline is defined as DIPAM of the corresponding non-injured finger of the contralateral hand.

The impact of an anti-adhesion treatment in patients with deep flexor tendon (FDP) injuries, as were recruited in this trial, is expected to be highest on the total active motion of the most distal finger joint (DIPAM), as its mobility is controlled solely by FDP. As expected, the DIPAM values of the injured digit increased over time in both treatment groups, accompanying hand recovery after surgery. At all time points measured (4, 6, 8, 12 weeks, and 6 and 12 months post-surgery/IMP administration), the DIPAM values were improved in patients in the PXL01 group compared to the patients in the placebo group, both in FAS and PPAS populations (Fig. 2). The difference between the groups, in favour of the PXL01 group, reached statistical significance at 6 months after surgery in the PPAS population (60 *vs.* 41 degrees in median values for PXL01 *vs.* placebo group, p = 0.016, rank analysis of covariance was applied as the assumption of normal distributed residuals was not fulfilled), while no statistically significant difference was observed in the FAS population. An exploratory statistical analysis using mixed model repeated measurement over all time points up to 12 months post-surgery confirmed a trend for improvement in the PXL01 group in the PPAS population (p = 0.060).

TAM2, defined as the sum of two finger joint ranges of motion (PIP and DIP joints), is the most frequently used measure to reflect hand recovery after surgery. TAM2 measured at 12 weeks post-surgery, which is the time-point when patients are first allowed to use their hand without restraints, was defined as the primary efficacy parameter of the trial. As expected, the TAM2 values of the injured digit increased over time in both treatment groups, accompanying hand recovery after surgery. From 12 weeks post-surgery and onwards, the patients treated with PXL01 presented improved TAM2 values as compared to the placebo group although the difference did not reach statistical significance by using rank analysis of covariance or mixed model repeated measurement analysis (Fig. S2).

**Figure 3:**
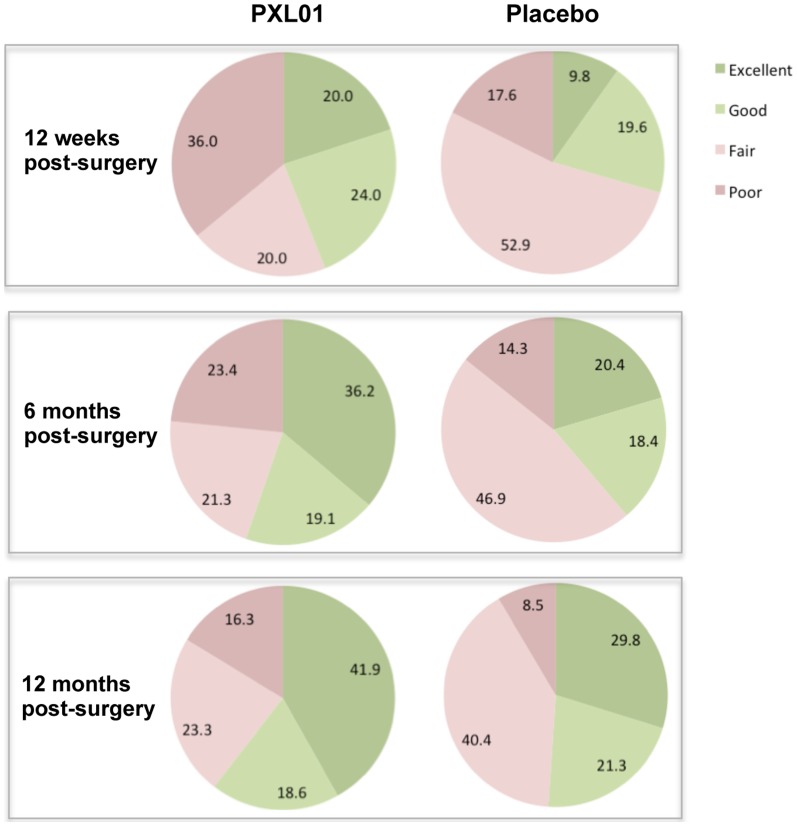
TAM2 absolute values of the injured finger graded according to Strickland's original classification at 12 weeks, 6 months and 12 months post-surgery/IMP administration in FAS. Mobility of ≥150 degress is assessed as “excellent”, 125 to 149 degrees as “good”, 90 to 124 degrees as “fair” and <90 degrees as “poor”. Values are numbers (percentages) of patients in each category.

TAM2 of the injured finger was also described according to Strickland's original categories, which is a frequently used classification system to describe functional hand recovery with ≥150 degrees of mobility assessed as “excellent”, 125 to 149 degrees as “good”, 90 to 124 degrees as “fair” and <90 degrees as “poor” [9]. This analysis, performed at 12 weeks as well as at 6 and 12 months post-surgery, indicated that the number of patients categorized as having excellent and excellent/good mobility was higher in the PXL01 group compared to the placebo group at all three time points, both is FAS and PPAS populations (Fig. 3). The difference between the groups, in favour of the PXL01 group, was most pronounced at 12 weeks and 6 months after surgery in the PPAS population (at 12 weeks: 46% of patients with excellent/good mobility in the PXL01 group compared to 29% in the placebo group, NS (p = 0.095 in chi-square analysis and p = 0.058 in generalized estimating equations analysis); at 6 months: 61% of patients with excellent/good mobility in the PXL01 group compared to 38% in the placebo group, p = 0.0499 in chi-square analysis, NS in generalized estimating equations analysis). No statistically significant differences were observed in the FAS population.

TAM3 is estimated as the sum of three finger joint ranges of motion (PIP, DIP and MCP joint). The mobility of the MCP joint is not influenced by peritendonous adhesions and therefore, TAM3 may be a less relevant readout of efficacy in this patient category. However, there was a tendency for the patients treated with PXL01 presenting higher TAM3 values as compared to the placebo group form 12 weeks post-surgery and onwards, although the difference did not reach statistical significance by using rank analysis of covariance or mixed model repeated measurement analysis (data not shown).

Consistently with measurements of total active motion, the patients in the PXL01 group, both is FAS and PPAS populations, presented shorter tip-to-crease distance at the visits performed at 6 and 12 months post-surgery/IMP administration, suggesting an improved recovery of hand function (Table 3). The improvement in PXL01 group, compared to placebo group, reached statistical significance at 6 months post-surgery in the PPAS population (5.0 *vs.* 15.5 mm in median values for PXL01 *vs.* placebo group, p = 0.048, rank analysis of variance was applied as the assumption of normal distributed residuals was not fulfilled). The difference did not reach statistical significance in the FAS population.

**Table 3:** Tip-to-crease distance of the injured finger at 6 and 12 months post-surgery by treatment.

	Visit		PXL01	Placebo
Tip-to-crease distance (mm) - FAS	6 months post-surgery	Mean (SD)	14.8 (16.9)	17.2 (14.7)
		Median (Min, Max)	10.0 (0, 60)	15.0 (0, 54)
	12 months post-surgery	Mean (SD)	9.7 (13.4)	13.1 (13.7)
		Median (Min, Max)	5.0 (0, 50)	10.0 (0, 56)
Tip-to-crease distance (mm) - PPAS	6 months post-surgery	Mean (SD)	11.8 (13.8)	17.5 (15.0)
		Median (Min, Max)	5.0 (0, 43)	15.5 (0, 54)
	12 months post-surgery	Mean (SD)	9.3 (12.8)	13.5 (13.8)
		Median (Min, Max)	0.8 (0, 45)	10.0 (0, 56)

FAS, full analysis set; PPAS, per protocol analysis set

**Figure 4:**
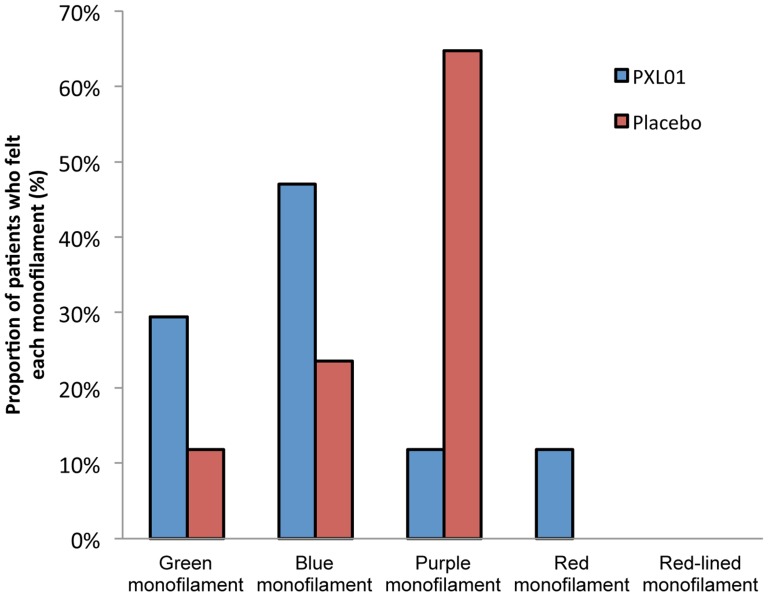
Sensory evaluation of the injured finger in patients with complete digital nerve injury at 12 weeks post-surgery/IMP administration in FAS: the first monofilament that a patient could feel. Values are numbers (percentages) of patients in each category.

Sensory evaluation was performed on patients with any complete digital nerve injury, independent on whether the radial or ulnar nerve was injured. The analysis at 12 weeks post-surgery was considered most relevant time point to indicate the nerve regeneration, with respect to axonal outgrowth after the nerve injury and repair. A higher proportion of these patients in the PXL01 group than in the placebo group could feel the thinnest monofilaments (green and blue) in FAS (PXL01 (n = 19): 74%, placebo (n = 17): 35%, p = 0.021 in chi-square analysis) as well as PPAS population (PXL01 (n = 17): 76%, placebo (n = 17): 35%, p = 0.016 in chi-square analysis) (Fig. 4).

At late visits, the investigator, or designee, judged whether the post-surgical mobility in the treated finger was impaired to the extent that the patient was recommended to go through tenolysis to improve the function. Although not statistically significant, by the final point of assessment, 12 months post-surgery, a higher proportion of patients in the placebo group were considered to likely benefit from tenolysis as compared to the patients in the PXL01 group (FAS: 28% *vs.* 16%, respectively, NS; PPAS: 30% *vs.* 12%, respectively, NS (p = 0.086); extended Cochran Mantel-Haenszel method of analysis). In addition, 2 patients in the placebo group and 1 patient in the PXL01 group had undergone tenolysis prior to the visit at 12 months post-surgery.

There were no statistically significant differences in grip strength comparing patients in the PXL01 group *vs.* placebo group (Table 4).

**Table 4:** Grip strength of the injured hand at 6 and 12 months post-surgery by treatment.

	Visit		PXL01	Placebo
Grip strength (kg) – FAS	6 months post-surgery	Mean (SD)	35.1 (12.4)	35.2 (12.8)
		Median (Min, Max)	36.7 (9, 67)	36.0 (10, 60)
	12 months post-surgery	Mean (SD)	41.2 (12.5)	40.3 (13.7)
		Median (Min, Max)	43.2 (12, 69)	39.7 (15, 68)
Grip strength (kg) – PPAS	6 months post-surgery	Mean (SD)	36.7 (11.7)	34.8 (13.4)
		Median (Min, Max)	37.7 (14, 67)	35.2 (10, 60)
	12 months post-surgery	Mean (SD)	41.8 (12.2)	40.2 (14.2)
		Median (Min, Max)	43.1 (12, 69)	39.7 (15, 68)

FAS, full analysis set; PPAS, per protocol analysis set.

### Safety evaluation

The frequency of SAEs was similar in both treatment groups (n = 11 events in the PXL01 group and n = 9 events in the placebo group reported by 15% and 13% of the patients, respectively). The majority (n = 11) of the SAEs were post-surgical ruptures of the repaired tendon reported by 5 patients in the PXL01 group and 6 patients in placebo group. The remaining SAEs were: adhesions resulting in tenolysis (PXL01: n = 1, placebo: n = 2), urticaria (PXL01: n = 1), procedural pain (PXL01: n = 1), syncope (placebo: n = 1), cerebrovascular accident (PXL01: n = 1), accidental death (PXL01: n = 1) and myeloproliferative disorder (PXL01: n = 1). Four SAEs (all tendon ruptures) were assessed as possibly related to treatment with IMP (PXL01: n = 3, placebo: n = 1). All other SAEs were assessed as unlikely related to IMP treatment.

The overall frequency of AEs was similar in both treatment groups (n = 73 events in the PXL01 group and n = 75 events in the placebo group reported by 62% and 57% of the patients, respectively). A majority of the AEs were judged as unlikely related to treatment with IMP. The proportion of patients reporting AEs assessed as possibly or probably related to treatment with IMP was similar in the PXL01 and placebo groups (14% *vs.* 9%), and there was no difference in the pattern of AEs assessed as possibly or probably related to treatment between the two groups. A vast majority of the AEs were mild to moderate in intensity (PXL01: 86%, placebo: 99%). The proportion of patients reporting AEs of severe intensity was higher in the PXL01 group compared to the placebo group (11% *vs.* 1%). None of the events with severe intensity, except for 1 event of tendon rupture in the PXL01 group, was assessed as possibly or probably related to treatment with IMP. Seven patients in PXL01 group (9%) and 8 patients in placebo group (11%) were withdrawn from the study due to AEs (11 due to tendon ruptures, 3 due to peritendon adhesions resulting in tenolysis, 1 due to accidental death). The most frequently reported AEs (reported by ≥5% of the patients in either or both treatment groups) were: nasopharyngitis (PXL01: 11%, placebo: 11%), tendon rupture (PXL01: 8%, placebo: 9%), peripheral oedema (PLX01: 8%, placebo: 6%), pain in extremity (PXL01: 3%, placebo: 9%), headache (PXL01: 6%, placebo: 3%) and localised infection (PXL01: 6%, placebo: 3%). Local signs of redness, pain, swelling and pruritus were infrequent and occurred with a similar incidence in both treatment groups. There were no abnormalities in mean vital signs values over time neither in the PXL01 group nor the placebo group.

## Discussion

Our randomized controlled clinical trial demonstrated that administration of 20 mg/ml PXL01 in 15 mg/ml sodium hyaluronate to patients admitted for surgical flexor tendon repair after hand injury was safe, well tolerated and did not interfere with tendon healing. Several efficacy parameters reflecting recovery of hand function after surgery - mobility of the most distal finger joint (DIPAM), TAM2 graded by Strickland's original classification system, tip-to-crease distance, sensory evaluation and frequency of tenolysis recommendations - indicated that PXL01 has benefits in this category of patients. The most pronounced difference between the PXL01 and placebo group, in favour of the PXL01 group, was observed at 6 months post-surgery/IMP administration in PPAS population. This was a first-in-patient phase II clinical trial with the main goal to generate the first data-based assessments of efficacy in a target population by testing a range of efficacy variables to identify potential beneficial outcomes. As such, it was decided not to make any adjustments for multiplicity in data analysis. Therefore, caution must be taken when interpreting the results from a battery of tests and larger clinical studies are required to confirm the beneficial effect of the treatment.

Postoperative rupture of the repaired tendon is the most serious complication after tendon repair surgery. Previously, at Skåne University Hospital (one of the clinical sites in this trial), the rupture rate was shown to be between 18 and 22% [8]. According to another publication, which summarizes the results reported in several different trials, the frequency of tendon rupture ranged from 4 to 14% [17]. In this trial, tendon rupture occurred in a similar proportion of patients in both treatment groups (PXL01: 8%, placebo: 9%) at a rate not higher than expected based on previous reports, suggesting that administration of the study product had no negative impact on tendon healing.

An increased range of motion in the most distal finger joint, combined with a decrease in tip-to-crease distance, as seen is this trial, is expected to facilitate the patients' ability to manipulate small items in their everyday life. Different questionnaires to evaluate this variable, such as the patients' opinion how the injury affected their activity of daily life, are available [18], and will be incorporated in future clinical trials.

Interestingly, the monofilament test at 12 weeks after surgery on the patients with a concomitant digital nerve injury showed that a higher proportion of the patients who were treated with PXL01 could feel the thinnest monofilaments, compared to those who received the placebo treatment. This indicates a better axonal outgrowth, which is important for prevention of target atrophy and neuronal cell death as well as aspects on cerebral plasticity [19]. This finding is interesting in view of the evidence that PXL01 inhibits plasminogen activator inhibitor type 1 (PAI-1) production [12]. The plasminogen activator system has shown to be beneficial for nerve regeneration *in vivo* [20]. In addition, PAI-1 is known to inhibit migration of Schwann cells from dorsal root ganglia *in vitro* [21]. Schwann cells are crucial for axonal outgrowth [22], [23] and by this mechanism PAI-1 may cause impairment in nerve regeneration. The inhibition of PAI-1 by PXL01 and data on improved nerve function in connection to flexor tendon repair surgery suggest PXL01 as a tentative drug also for stimulation of axonal outgrowth after nerve injury and repair.

In this trial, the differences in total active motion and tip-to-crease distance comparing the PXL01 and placebo group of patients, in favour of the PXL01 treatment, reached statistical significance at 6 months post-surgery. This is consistent with the evidence showing that the inflammatory phase of adhesion formation is initiated shortly after the surgery, while the maturation and remodelling of adhesions proceeds for at least 9 months post-surgery [24], [25]. Moreover, due to the risk for tendon rupture, the patients are advised not to use their hand without restrictions during the first 12 weeks post-surgery. Therefore, active use of the hand after these first 12 weeks is likely to further improve the mobility primarily in patients with no or limited amount of peritendinous adhesions, and correspondingly, to amplify the differences between the treatment groups.

Prior this trial, the results of prospective randomized clinical trials in flexor tendon repair surgery for two different anti-adhesion products have been published. ADCON-T/N, a bioresorbable gel composed of gelatine and a carbohydrate polymer in phosphate buffered saline, was shown to have no benefit in one trial [26], while advantage in post-surgical finger mobility was reported in the second clinical trial [27] in zone II flexor tendon repair. However, there was a risk for significant disadvantages as increased delayed rupture rate of the repaired tendon was reported in the ADCON group [28]. A trial with native sodium hyaluronte treatment in connection to flexor tendon repair in zone II did not show any improvement in clinical outcome [6]. Thus, to our knowledge, this is the first report of safe and effective anti-adhesion treatment in surgical repair of flexor tendon injuries.

In this study, statistically significant and clinically relevant improvement in hand function in patients treated with PXL01, compared to the placebo group of patients, was detected for several efficacy parameters in PPAS. Several factors may have contributed to the fact that statistical significance was not reached in FAS population. A large number of patients in the FAS had extensive deviations in visit windows, which is the main limitation of the study. The main analysis of the efficacy variables in this trial was based on visit number rather than actual visit date, and therefore, it is difficult to demonstrate statistical significance for these parameters, which all change over time, in FAS. In addition, analysis of injury characteristics in the two treatment groups revealed a number of differences, which might have compromised the data quality in favour of the placebo treatment. A higher proportion of patients in the PXL01 group had multiple digit injuries (PXL01: 36%, placebo: 24%) and were treated for the injury to the little finger (PXL01: 55%, placebo 43%). Several reports indicate that a less satisfactory hand recovery is expected if multiple fingers in the same hand are injured [29] as well as for injuries to the little finger as compared to the other fingers [30], [31], after flexor tendon repair. Moreover, the relative proportion of patients without an injury to the FDS tendon in the treated digit was slightly lower in the PXL01 group (PXL01: 28%, placebo: 35%). While only the FDP tendon controls to the mobility of the DIP joint, both FDS and FDP contribute to the mobility of the PIP joint. Thus, the measurements of TAM2, TAM3 as well as grip strength might have been influenced by this difference in the favour of placebo treatment.

Our previous studies have shown that several administrations of water solution of PXL01 in rats shortly after abdominal surgery improved the anti-adhesive properties of the peptide, compared with the single treatment, indicating that repeated administrations or slow duration of the drug release would be beneficial [12]. Since single administration is preferable in clinical setting, a high molecular weight sodium hyaluronate was chosen as a carrier for PXL01 in this clinical trial as it has previously been shown to provide controlled release of the peptide [12]. Moreover, PXL01 is readily soluble and sufficiently stable in sodium hyaluronate, and the PXL01-containing sodium hyaluronate hydrogel is bioadhesive and easy to apply to the surgical area. Importantly, the carrier high molecular weight sodium hyaluronate has been shown to exert anti-inflammatory effect by influencing a variety of immune cell functions and reducing the concentration of inflammatory mediators [32]–[36], and a number of studies have addressed the ability of sodium hyaluronate alone to prevent adhesion formation around flexor tendons. While no improvement was reported in some of these studies [6], [37], [38], a chemically modified carbodiimide derivatized sodium hyaluronate in combination with gelatin and/or lubricin was recently shown to significantly reduce gliding resistance and decrease peritendinous adhesion formation, although the treatment was also associated with an impaired tendon healing strength [39]–[43]. Our previous studies in a rabbit model of flexor tendon repair surgery showed that PXL01 in sodium hyaluronate significantly improved the digit mobility compared with the treatment with sodium hyaluronate alone, while the sodium hyaluronate group was not different from the sham-operated digits [13]. However, the design of this clinical trial does not allow to conclude whether sodium hyaluronate might have contributed to the anti-adhesive properties of the treatment. To address this question, one treatment arm with sodium hyaluronate alone will be incorporated into the future clinical trials.

A major strength of this study is its prospective and randomized design, application of the most up-to-date surgical techniques and rehabilitation protocols and careful and long follow-up of the patients. We believe the results of the study are generalizable for adhesion prevention in several additional indications involving surgery on tendons and nerves in hand and lower arm, and will provide a basis for investigation of potential applicability of PXL01 in extensor and flexor tendon injuries, tenolysis, tendon transfers and transplantations, complex injuries with fractures and even various nerve repair and reconstruction procedures.

In conlusion, the current study suggests that treatment with the peptide PXL01, formulated with native sodium hyaluronate carrier, in connection to the surgical flexor tendon repair after hand injury, improves the clinical outcome in terms of mobility of the affected finger. A potential for a favourable role of PXL01 in sodium hyaluronate to stimulate nerve regeneration is also raised. Confirmatory clinical trials are warranted to provide further evidence for safety and efficacy of PXL01 as well as to determine the most efficient dose and the health economic benefits of the treatment.

## Supporting Information

Figure S1 **Efficacy endpoints.**
(TIF)

Figure S2 **TAM2 over time.**
(TIF)

Table S1 **The demographics.**
(DOCX)

Table S2 **The baseline characteristics.**
(DOCX)

Checklist S1 **CONSORT checklist.**
(DOC)

Protocol S1 **Study protocol.**
(PDF)
