# Supplementary figures and images for: PXL01 in Sodium Hyaluronate for Improvement of Hand Recovery after Flexor Tendon Repair Surgery: Randomized Controlled Trial
Source: PLoS One. 2014 Oct 23;9(10):e110735. doi: 10.1371/journal.pone.0110735 (PMC4207831; doi:10.1371/journal.pone.0110735)

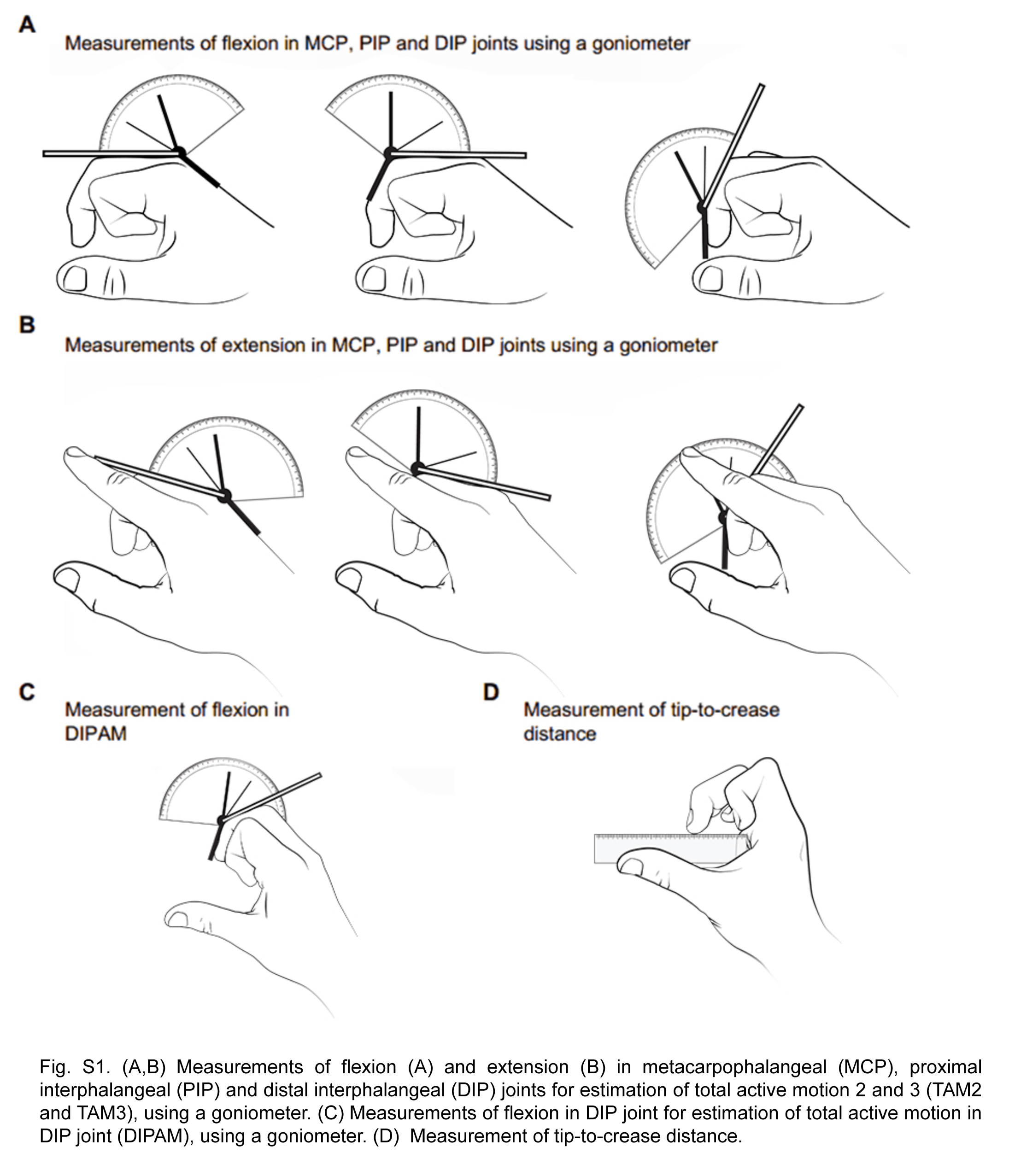

Supplement: Figure S1 — Efficacy endpoints. (TIF) [file pone.0110735.s001.tif]

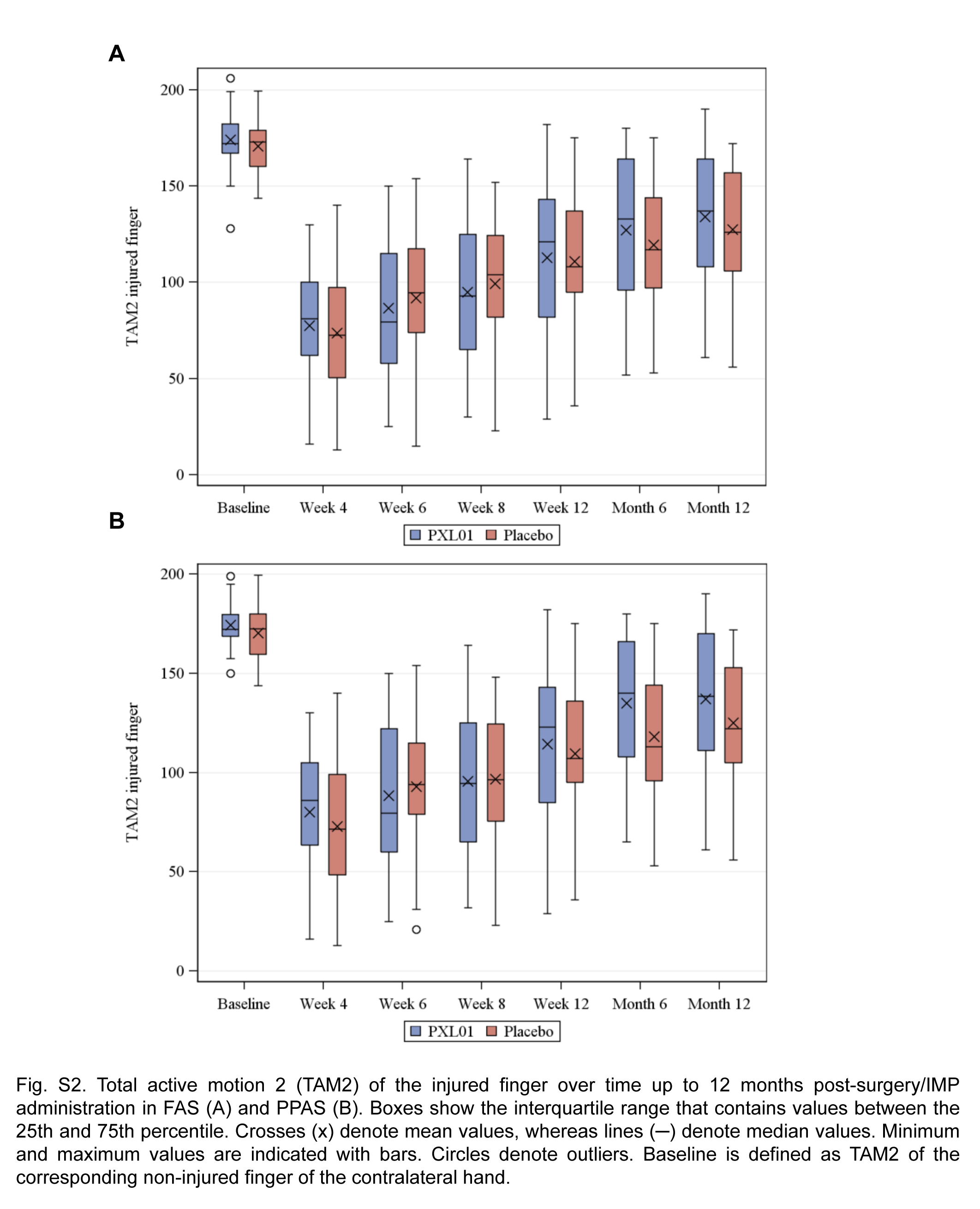

Supplement: Figure S2 — TAM2 over time. (TIF) [file pone.0110735.s002.tif]
