# Supplementary material for: PXL01 in Sodium Hyaluronate for Improvement of Hand Recovery after Flexor Tendon Repair Surgery: Randomized Controlled Trial
Source: PLoS One. 2014 Oct 23;9(10):e110735. doi: 10.1371/journal.pone.0110735 (PMC4207831; doi:10.1371/journal.pone.0110735)
Supplement: Table S1 — The demographics. (DOCX) [file pone.0110735.s003.docx]

Table S1. The demographics of the patients*

|  |  | **PXL01** | **Placebo** | **All** |
| --- | --- | --- | --- | --- |
| Age (years) | n/nmiss | 64/0 | 68/0 | 132/0 |
|  | Mean (SD) | 35.5 (13.6) | 36.2 (15.0) | 35.9 (14.3) |
|  | Q1, Q3 | 23.5, 48.0 | 24.5, 49.0 | 24.0, 48.5 |
|  | Median (Min, Max) | 34.5 (12, 66) | 32.0 (16, 68) | 33.0 (12, 68) |
| Sex | Male | 47 (73.4%) | 49 (72.1%) | 96 (72.7%) |
|  | Female | 17 (26.6%) | 19 (27.9%) | 36 (27.3%) |
| Race | White | 61 (95.3%) | 62 (91.2%) | 123 (93.2%) |
|  | Asian or Pacific Islander | 0 | 2 (2.9%) | 2 (1.5%) |
|  | African descent | 1 (1.6%) | 1 (1.5%) | 2 (1.5%) |
|  | Mixed/multi-racial | 1 (1.6%) | 0 | 1 (0.8%) |
|  | Other | 1 (1.6%) | 3 (4.4%) | 4 (3.0%) |

*Percentages are based on the number of patients in the Full Analysis Set (FAS)
