# Supplementary material for: PXL01 in Sodium Hyaluronate for Improvement of Hand Recovery after Flexor Tendon Repair Surgery: Randomized Controlled Trial
Source: PLoS One. 2014 Oct 23;9(10):e110735. doi: 10.1371/journal.pone.0110735 (PMC4207831; doi:10.1371/journal.pone.0110735)
Supplement: Table S2 — The baseline characteristics. (DOCX) [file pone.0110735.s004.docx]

Table S2. Summary of injury baseline values*

|  |  | **PXL01** | **Placebo** | **All** |
| --- | --- | --- | --- | --- |
| Is the dominant hand injured? | Yes | 28 (43.8%) | 31 (45.6%) | 132/0 |
| Type of trauma? | Sharp | 59 (92.2%) | 61 (89.7%) | 120 (90.9%) |
|  | Blunt | 2 (3.1%) | 1 (1.5%) | 3 (2.3%) |
|  | Machinery type | 3 (4.7%) | 6 (8.8%) | 9 (6.8%) |
| Nature of wound? | Other | 2 (3.1%) | 0 | 2 (1.5%) |
|  | Tidy | 55 (85.9%) | 62 (91.2%) | 117 (88.6%) |
|  | Untidy | 7 (10.9%) | 6 (8.8%) | 13 (9.8%) |
| Digits with complete division of FDP in zone I or zone II? | Digit 2 | 15 (23.4%) | 19 (27.9%) | 34 (25.8%) |
|  | Digit 3 | 10 (15.6%) | 10 (14.7%) | 20 (15.2%) |
|  | Digit 4 | 13 (20.3%) | 14 (20.6%) | 27 (20.5%) |
|  | Digit 5 | 35 (54.7%) | 29 (42.6%) | 64 (48.5%) |
| FDS injury in zone I or zone II of the treated digit? | None | 18 (28.1%) | 24 (35.3%) | 42 (31.8%) |
|  | Partial Division | 17 (26.6%) | 18 (26.5%) | 35 (26.5%) |
|  | Complete Division | 29 (45.3%) | 26 (38.2%) | 55 (41.7%) |
| Multiple digit injures | Yes | 23 (35.9%) | 16 (23.5%) | 39 (29.5%) |
| Skin injuries | Other | 0 | 2 (2.9%) | 2 (1.5%) |
|  | No loss | 64 (100.0%) | 66 (97.1%) | 130 (98.5%) |
|  | Loss requiring graft | 0 | 0 | 0 |
|  | Loss requiring flap | 0 | 0 | 0 |
| Circulation injuries | Other | 0 | 5 (7.4%) | 5 (3.8%) |
|  | Intact | 64 (100.0%) | 63 (92.6%) | 127 (96.2%) |

*Percentages are based on the number of patients in the Full Analysis Set (FAS). FDP, Flexor Digitorum Profundus; FDS, Flexor Digitorum Superficialis
