# Supplementary material for: PXL01 in Sodium Hyaluronate for Improvement of Hand Recovery after Flexor Tendon Repair Surgery: Randomized Controlled Trial
Source: PLoS One. 2014 Oct 23;9(10):e110735. doi: 10.1371/journal.pone.0110735 (PMC4207831; doi:10.1371/journal.pone.0110735)
Supplement: Protocol S1 — Study protocol. (PDF) [file pone.0110735.s006.pdf]

CLINICAL STUDY PROTOCOL

A prospective double-blind, randomised concept study of PXL01 versus placebo in flexor tendon surgery

**Protocol Number**	PHSU02
**Protocol Version**	Version 3, 2010-06-16
**Protocol Status**	Final
**Products**	PXL01 and placebo
**Phase**	II
**EudraCT No**	2009-012703-25
**Lead Principal Investigator**	Monica Wiig, MD, PhD<br>Dept. of Hand Surgery<br>Uppsala University Hospital, ing 70<br>SE-751 85 Uppsala, Sweden
**Sponsor**	PharmaSurgics in Sweden AB (PSAB)<br>Arvid Wallgrens Backe 20<br>SE-413 46 Göteborg, Sweden
**Study Management**	Pharma Consulting Group AB (PCG)<br>Kungsängsvägen 19<br>SE-753 23 Uppsala, Sweden
**Planned clinical start**	Q4 2009
**Planned clinical completion**	Q1 2012

LIST OF ABBREVIATIONS AND DEFINITION OF TERMS

Abbreviation, acronym or specialist term	Explanation
AE	Adverse Event
ATC	Anatomic Therapeutic Chemical (classification of drugs)
CA	Competent Authorities
CSP	Clinical Study Protocol
DASH	Disabilities of the Arm, Shoulder, and Hand
DIP	Distal Interphalangeal joint
DIPAM	Active Motion in the DIP joint
e-CRF	Electronic Case Report Form
FAS	Full Analysis Set
FDP	Flexor Digitorium Profundus
FDS	Flexor Digitorium Superficialis
GCP	Good Clinical Practice
IB	Investigator’s Brochure
ICMJE	International Committee of Medical Journal Editors
IEC	Independent Ethics Committee
IMP	Investigational Medicinal Product
iv	Intravenous
MCP	Metacarpophalangeal joint
MedDRA	Medical Dictionary for Regulatory Activities
NSAID	Non-steroidal anti-inflammatory drug
PSAB	PharmaSurgics AB
PCG	Pharma Consulting Group AB
PIP	Proximal Interphalangeal joint
PPAS	Per Protocol Analysis Set
QoL	Quality of Life
SADR	Serious Adverse Drug Reaction
SAE	Serious Adverse Event
SD	Standard Deviation

**Abbreviation, acronym or specialist term**	**Explanation**
TAM	Total Active Motion
TAM2	Total Active Motion (PIP, DIP)
TAM3	Total Active Motion (MCP, PIP, DIP)
TPM	Total Passive Motion
TPM2	Total Passive Motion (PIP, DIP)
TPM3	Total Passive Motion (MCP, PIP, DIP)
U.S.P.	United States Pharmacopeia
Zone I	Extends from the second phalanx where the FDS is inserted into the bone to the distal end of the third phalanx
Zone II	Extends from the distal palmar fold to the second phalanx where the FDS is inserted into the bone

STUDY PROTOCOL IN SHORT

Study title	A prospective double-blind, randomised concept study of PXL01 versus placebo in flexor tendon surgery
Protocol number	PHSU02
EudraCT number	2009-012703-25
Phase of development	Phase II
Name and address of Sponsor	PharmaSurgics AB<br>Arvid Wallgrens Backe 20<br>SE-413 46 Göteborg, Sweden
Lead Principal Investigator	Monica Wiig, MD, PhD<br>Dept. of Hand Surgery<br>Uppsala University Hospital, ing 70<br>SE-751 85 Uppsala, Sweden
Investigational medicinal product, dose, and administration	PXL01 is a synthetic peptide sequentially derived from human lactoferrin. PXL01 20 mg/ml is formulated in a viscous solution of 15 mg/ml sodium hyaluronate. The drug product is administered locally between the flexor tendon and the tendon sheath and around the tendon sheath at a volume of 0.5 ml. Administration of the product is carried out after repair of the flexor tendon but before closure of the surgical wound.
Name of active ingredient	PXL01
Number of patients	According to the patient number calculations, 110 evaluable patients are needed. To adjust for withdrawals, 138 patients will be included in the study. A recalculation of the patient number will be made in connection to the planned blinded interim analysis, which will be performed when 69 patients (50%) have completed the 12-week visit. If the calculations suggest a patient number adjustment, a study protocol amendment will be submitted to the MPA.
Number and name of study sites	The following three sites will include the patients in this study:<br>1. Monica Wiig, Dept. of Hand Surgery, Uppsala University Hospital, Uppsala, Sweden<br>2. Lars Dahlin, Dept. of Hand Surgery, Malmö University Hospital, Malmö, Sweden<br>3. Jan Fridén, Dept. of Hand Surgery, Sahlgrenska University Hospital, Gothenburg, Sweden<br>4. Lars Hagberg, Dept. of Hand Surgery, Södersjukhuset, Stockholm, Sweden
Objectives	The objectives of the study are to assess efficacy, safety, and

	handling of PXL01 in patients with flexor tendon injury in zone I or II.
Assessment variables	<u>Efficacy variables:</u><br>• Total Active Motion based on PIP and DIP joints (TAM2) (primary variable at 12 weeks after surgery/IMP administration)<br>• TAM2 graded according to Strickland's original classification system<br>• TAM2 over time<br>• TAM3 (TAM based on MCP, PIP, and DIP joints)<br>• TAM3 over time<br>• Total Active Motion in DIP joint (DIPAM)<br>• Grip strength<br>• Tip-to-crease distance<br>• Disabilities of the Arm, Hand, and Shoulder (DASH) Quality of Life (QoL)<br>• Sensory evaluation with Semmes-Weinstein monofilaments (only for patients with any complete digital nerve injury)<br>• Recommendation for tenolysis<br>• Total Passive Motion (TPM2 and TPM3)<br><br><u>Safety variables:</u><br>• The frequency and severity of Adverse Events (AEs)<br>• Vital signs (blood pressure, heart rate, temperature)<br>• Blood safety (clinical chemistry, haematology)<br>• Observation of the surgical area<br>• Rupture rate<br><br><u>Handling variables:</u><br>• Handling- and administration questionnaire
Study design and procedures	This is a multi-centre, randomised, double-blind, parallel study comparing PXL01 and placebo. Patients admitted for flexor tendon repair in zone I or II are screened for inclusion.<br>The patients will undergo 7 visits within the time frame of the study. In addition, two follow-up visits will be performed.<br><u>Visit 1:</u> Screening/Surgery/IMP administration/post-surgery (Informed consent, demography, medical history, general injury characteristics, pregnancy test, vital signs, safety laboratory, eligibility criteria, specific injury characteristics, surgery, randomisation, IMP administration, concomitant medications, AE/SAE)<br><u>Visit 2:</u> 1-5 days after IMP administration (QoL, mobilisation excercises, concomitant medications, AE/SAE)<br><u>Visit 3:</u> 2 weeks after IMP administration (vital signs, safety laboratory, examination of the surgical area, tendon rupture, mobilisation excercises, mobilisation compliance, concomitant

	medications, AE/SAE)<br><br><u>Visit 4:</u> 4 weeks after IMP administration (examination of the surgical area, TAM, DIPAM, tip-to-crease, TPM, tendon rupture, mobilisation excercises, mobilisation compliance, concomitant medications, AE/SAE)<br><br><u>Visit 5:</u> 6 weeks after IMP administration *(see Visit 4)*<br><br><u>Visit 6:</u> 8 weeks after IMP administration (examination of the surgical area, TAM, DIPAM, tip-to-crease, TPM, tendon rupture, mobilisation excercises, mobilisation compliance, concomitant medications, AE/SAE)<br><br><u>Visit 7:</u> 12 weeks after IMP administration (QoL, examination of the surgical area, TAM, DIPAM, tip-to-crease, TPM, sensory evaluation, tendon rupture, recommendation for tenolysis, mobilisation compliance, concomitant medications, AE/SAE)<br><br><u>Follow-up 1:</u> 6 months after IMP administration (QoL, TAM, DIPAM, tip-to-crease, grip strength, TPM, sensory evaluation, tendon rupture, recommendation for tenolysis, concomitant medications, AE/SAE)<br><br><u>Follow-up 2</u>: 12 months after IMP administration (QoL, TAM, DIPAM, tip-to-crease, grip strength, TPM, sensory evaluation, tendon rupture, recommendation for tenolysis, concomitant medications, AE/SAE)<br><br>Before any study-related assessment takes place, patients are given verbal and written information about the study and informed consent is achieved from the patient and/or legal guardian. The study should be carried out in accordance with the Clinical Study Protocol (CSP), ICH guidelines for Good Clinical Practise (GCP), and applicable regulatory requirements.
Inclusion criteria	1. Complete division of flexor digitorium profundus tendon (FDP) in zone I or II, with or without division of the flexor digitorium superficialis (FDS) and possible to rejoin with tendon suture<br>2. Open flexor tendon injury sutured within 14 days after trauma<br>3. 12-75 years of age<br>4. Signed informed consent prior to any study related procedures

Exclusion criteria	1. Treatment with any investigational product within 4 weeks of study entry<br>2. Patients previously included in the study<br>3. Thumbs with complete or partial division of flexor pollicis longus (FPL)<br>4. Concomitant fracture(s) requiring immobilisation<br>5. Injuries with associated soft tissue loss<br>6. Severe crush injury<br>7. Palmar plate injury requiring immobilisation<br>8. Devascularisation/requirement of vascular repair<br>9. Joint injuries<br>10. Bilateral injuries<br>11. Previous flexor tendon surgery in the digit, which is to be treated wih IMP<br>12. Reduced motion in the digit, which is to be treated with IMP, or the corresponding digit prior to the injury<br>13. Compliance with mobilisation protocol not expected<br>14. Alcohol or drug abuse<br>15. Severe intercurrent illness, which in the opinion of the Investigator, may put the patient at risk when participating in the study, or affect the patient's ability to take part in the study<br>16. Pregnant or lactating females<br>17. Fertile women who do not accept the consistent and correct use of highly effective methods of birth control defined as implants, injectables, combined oral contraceptives, intra-uterine device (IUD)s, sexual abstinence or vasectomised partner during the first two weeks post-surgery. A condom alone is not considered an acceptable method for birth control, not even together with spermicide.<br>18. Known allergy to any component of the study product or placebo<br>19. Patients suffering from:<br>• Diabetes Mellitus patients where significant diabetic complications may delay healing according to the investigator's judgement<br>• Rheumatoid arthritis<br>20. Or patients treated with:<br>• Systemic steroids within one month<br>• Immunosuppressive drugs within three months<br>• Daily use of NSAIDs within one week or occasional use within 8 hours
Statistical methods	<u>Analysis sets:</u><br>The full analysis set (FAS) for efficacy analyses will include all patients who have completed the flexor tendon surgery and received the study medication.<br>The per protocol analysis set after 12 weeks (3 months) (PPAS3m) is a subset of the FAS and will include all patients in the FAS who have completed the study until the 12-week visit with no major protocol violations.

	The per protocol analysis set after 12 months (PPAS12m) is a subset of the FAS and will include all patients in the FAS who have completed the study until the last follow-up visit at 12 months with no major protocol violations.<br><br>The safety analysis set (Safety) for safety analyses will include all patients who have completed the flexor tendon surgery and received the study medication.<br><br><u>Primary objective:</u><br><br>Analysis of TAM2 at 12-week visit.<br><br>The null hypothesis to be tested is that there is no difference in TAM2 at 12 weeks after surgical procedure between patients treated with PXL01 and patients treated with placebo. The alternative hypothesis is that there is a difference between treatments.<br><br>The primary objective will be tested using analysis of covariance (ANCOVA) with TAM at 12 weeks and the corresponding TAM of the contralateral hand as baseline and also including factors for centre, treatment and time between open flexor tendon injury and surgery (-4 days, >4 days).<br><br><u>Secondary objectives:</u><br><br>The following secondary objectives will be analysed in a similar way as the primary objective:<br><br>• TAM2 at all time points except for 12 weeks<br>• TAM3 at all time points<br>• Total Active Motion in DIP joint (DIPAM)<br>• Grip strength<br>• Total Passive Motion (TPM2 and TPM3)<br><br>TAM2 and TAM3 over time will be analysed using a repeated measurements approach.<br><br>For tip-to-crease distance analysis of variance (ANOVA) including centre, treatment and time between open flexor tendon injury and surgery (-4 days, >4 days) as factors will be used.<br><br>For tenolysis recommendation Extended Mantel-Haenszel method including centre, treatment and time between open flexor tendon injury and surgery (-4 days, >4 days) will be used.<br><br>The DASH QoL will be analysed by Wilcoxon rank-sum test.<br><br>The following secondary objectives will be presented descriptively only:<br><br>• TAM2 graded according to Strickland's Original Classification System<br>• Sensory evaluation with Semmes-Weinstein monofilaments<br><br><u>Safety variables:</u>

	For rupture rate Extended Mantel-Haenszel method including centre, treatment and time between open flexor tendon injury and surgery (-4 days, >4 days) will be used.<br>All other safety variables will be presented descriptively only.
Study reporting	After all patients have completed the 12-week visit, a report on blinded data will be produced. Only groups of patients will be displayed and no treatment code will be revealed in the report. The analyses will be performed by a study independent statistician. Meanwhile, the study will continue until all patients have completed the 12-month visit. After the last follow-up at 12 months, a more substantial report will be written.
Planned study time table	First Patient enrolled Q4 2009<br>Last Patient out Q1 2012
